# The perceived effects and comfort of various body armour systems on police officers while performing occupational tasks

**DOI:** 10.1186/s40557-018-0228-x

**Published:** 2018-02-28

**Authors:** B. Schram, B. Hinton, R. Orr, R. Pope, G. Norris

**Affiliations:** 1 Tactical Research Unit, Bond University, Robina, Australia; 2 NSW Police - Health and Fitness Unit, Sydney, Australia; 3 Charles Sturt University, Albury, Australia; 4 NSW Police - Operational Safety and Skills Command, Robina, Australia

**Keywords:** Light armour, Personal protective equipment, Load, Law enforcement

## Abstract

**Background:**

The nature of police work often necessitates use of Individual Light Armour Vests (ILAVs) for officer protection. Previous research has demonstrated various biomechanical and physical performance impacts of ILAVs, however, little knowledge exists on the individual officer’s perceptions of ILAV. The aim of this study was to investigate officers’ perceptions of the impacts of three different ILAVs and normal station wear whilst performing police occupational tasks.

**Methods:**

A prospective, within subjects, repeated measures design was employed in which 11 serving police officers wore each of three different types of body armour (ILAV A, ILAV B or ILAV C) and normal station wear for a full day while performing tasks including a simulated victim drag, a patrol vehicle exit and a marksmanship shoot. Ratings of Perceived Exertion (RPE) and a Visual Analogue Scale (VAS; − 10 to + 10) were used to examine officer perceptions of each ILAV. Finally, officers were asked to indicate areas of both discomfort and comfort of each ILAV on a mannequin chart.

**Results:**

Officers perceived less effort was required for the victim drag whilst wearing ILAV B (RPE = 3.6/10) when compared to ILAV A, ILAV C and even station wear (RPE = 4.7/10, 4.0/10, 3.8/10, respectively). A positive impact on performance was perceived for ILAV B (VAS = + 0.26) when performing a patrol vehicle exit and sprint task but not for the other two ILAVs (VAS = − 3.58, − 0.55, − 0.85, respectively). Officers perceived a positive impact of ILAV B (VAS = + 2.7) and station wear (VAS = + 1.4) and a negative impact of ILAVs A and C (VAS = − 2.1, − 1.7 respectively) on marksmanship. Despite all armour types being criticized for discomfort, ILAV B received lower ratings of discomfort overall, and some positive comments regarding both comfort and performance.

**Conclusions:**

Officers perceived ILAV B to have positive effects on task performance. It was also rated more comfortable than the other two, possibly due to a longer torso design which shifted load from the shoulders to the hips and pelvis. Officer perceptions of comfort and effects on occupational performance should be considered when designing and procuring armour systems. Although ILAVs may be similar, perceived impacts may vary between officers.

## Background

Policing duties may intermittently involve periods of running, jumping, crawling and engaging in combat without warning [1, 2]. Numerous tactical occupations, including military and law enforcement, utilise body armour to improve survivability and protect against stabbing injuries [3, 4]. Despite these benefits, any additional loads carried by tactical personnel may detrimentally affect the carrier’s mobility, reduce their operational capability, and lead to various musculoskeletal injuries [5–7].

More recently, lighter Individual Light Armour Vests (ILAV) weighing between 2.7–3.8 kg have been implemented to reduce officer fatalities from stabbing, blunt trauma and small calibre bullets [3]. However, prior to the implementation of any ILAV for tactical populations, the potential decrements they cause in performance of occupational tasks and the degree to which they are acceptable to the wearer must be scrutinised. A balance between protection, physical restriction and performance impacts during conduct of tactical duties must be achieved, and officers must perceive the ILAV to be acceptable. Previous studies have identified higher ratings of perceived exertion (RPE) whilst shooting, crawling, lifting and exercising while wearing armour, and greater levels of discomfort when wearing heavier loads (6–27 kg) [8–12].

The importance of acceptance of additional loads by the tactical personnel who must carry them was evident in a study by Ramstrand et al. [13], who examined the subjective responses of police officers who were provided with a load bearing vest in place of their standard utility belt. Despite the load bearing vest being rated on average to be more comfortable than the utility belt, 33% of participants reported they would not choose to wear a load bearing vest in the future, if given the option, due to discomfort and decreased range of motion [13].

Given the importance of acceptance by the wearer before implementation of ILAVs, the opinions of individuals required to wear the equipment should be sought during the ILAV design or procurement phases, if user engagement with the proposed ILAVs is to be achieved. Therefore, the aim of this study was to investigate officers’ perceptions of the comparative impacts of three different types of ILAV and normal station wear when they were worn while performing occupational tasks associated with policing duties.

## Methods

### Study design

A prospective, within-subjects, repeated measures design was employed, using a counterbalanced randomization procedure to determine the order of types of ILAV and normal station wear worn by each participating officer. Each officer served as their own control and wore each of three types of ILAV for one full day, and normal station wear for one full day. On each day, all measures were taken at the same time of day while the officers wore the ILAV or standard station wear assigned for that particular day. The counterbalanced randomization procedure controlled for any potential activity learning effects and other factors (eg ambient conditions) that might have varied across the 4 days of data collection.

### Participants

Data collection for the study was conducted at an Australian State police college in 2016. Eleven research volunteer officers who were all qualified and serving members of the Australian State Police Force, served as participants for this study. The officer’s characteristics can be seen in Table 1 below.

**Table 1:** Participants Characteristics. Expressed as mean (SD)

	Age (years)	Weight (kg)	Height (cm)	Length of Service (months)
Males (*n* = 6)	40 ± 8	83 ± 20	177 ± 9	78 ± 12
Females (*n* = 5)	27 ± 3	68 ± 18	164 ± 7	92 ± 9
Group (*n* = 11)	34 ± 9	76 ± 20	171 ± 10	65 ± 4

To improve the translation of this research across the general State police force population, equal numbers of female and male officers were initially recruited, with two participants per gender sized as each of small, medium and large, with respect to the standard sizes used by the ILAV suppliers. This process and diversification allowed for the comparison of the perceived impacts of three ILAV and normal station wear in relation to both gender and body size. All of the 12 initially-recruited officers were provided an initial briefing regarding the program and, if they expressed willingness to participate (as they all did), they were invited to provide written informed consent for participation. One recruited female officer was unable to commence the research due to medical concerns which were identified at this stage and so the final sample was reduced to 11 officer participants. All participants formally consented to participation and the study was approved by the Bond University Human Research Ethics Committee (protocol number 15803).

### ILAV types

The three different types of ILAV (types A, B and C) were all weighed (Tanita, BF-679 W) prior to provision. Each type of ILAV was then fitted with standard equipment worn by each officer as part of their normal duties. This equipment was individualised and varied slightly due to each officer’s daily taskings and preferences. This equipment carried as part of normal duties also made up the fourth condition; normal station wear (N). On the days of testing, officers wore their allocated load configuration for the duration of the day. Officers were randomly allocated to one of four initial conditions (ILAV A, B or C or normal station wear (N) using a lot draw on the first day of data capture which employed a counterbalanced approach. Following the first day of data collection, each participant progressed to the next ILAV to be trialled, in the specified sequence, which progressed from ILAV A to ILAV B to ILAV C to normal station wear to ILAV A.

### Data capture procedure

To minimise any diurnal variations a standardised program was followed on each day. Noting that this program of data capture was part of a larger project, with other measures also captured, the aspects of the daily program relevant to this study are shown in Table 2, and each listed activity is further discussed in the sections that follow.

**Table 2:** Daily sequence of events

Time	Measure	Activity
08:00		Morning brief and allocation of ILAV and equipment issue and testing
09:30	RPE Scale	Victim drag
11:00	VAS Scale	Rapid Patrol Vehicle Exit and 5 m Sprint
14:45	VAS Scale	Marksmanship task
16:30	Subjective evaluation	Daily debrief

### RPE scale - 10 m victim drag

The Rating of Perceived Exertion (RPE; also known as the Perceived Rate of Exertion [PRE] or Borg scale) is a measure of a person’s perception of the rate of effort required for a given task. While the original scale was rated between a score of 6 (no exertion) and 20 (maximal exertion) [14], the modified scale used in this study ranges from a score of 1 to 10 [14]. To aid officers in determining their perceived level of effort, descriptive terms were included beside some of the numbers in the RPE scale, to act as a guide. Officers were asked to rate their perceived level of effort during the 10 m victim drag task (described below), immediately on finishing the task.

The 10 m victim drag scenario utilized a mannequin fitted with a ballistic vest (80 kg). Officers were required to lift the shoulders of the mannequin off the ground and, moving backward, drag the mannequin 6 m, complete a 90-degree right hand turn through a doorway, and continue to drag the mannequin another 4 m to the end of the track. This configuration was designed to mimic retrieving a victim from the centre of a road and then dragging them back and behind cover. The distances officers covered for the victim drag task were measured using a digital mini-measuring wheel (Senshin Industry Co., Ltd. Osaka: Japan). Officers were allowed an initial practice run at their estimated 80% of maximum capacity, to familiarize themselves with the scenario and as a warm up.

### VAS - rapid patrol vehicle exit and marksmanship tasks

The Visual Analogue Scale (VAS) is a commonly used tool to quantify perceptions of a particular type of experience [15]. On completion of two key tasks (rapid patrol vehicle exit and marksmanship shoot) a VAS was used to subjectively measure how the officers felt the ILAVs they were wearing impacted on their performance of that task in either a positive or negative way or if indeed it had no impact at all. The officer was asked to draw a vertical line anywhere along the 20 cm scale (− 10 cm indicating the ILAV had a negative impact to + 10 cm indicating the ILAV made the task easier). The distance from center (‘no change’) was measured and recorded in mm.

**Fig. 1:**
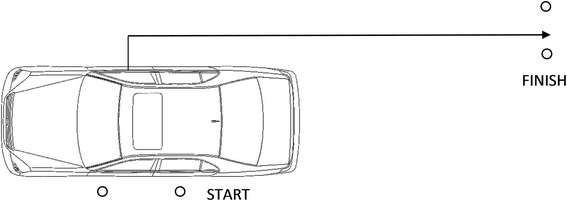
Patrol vehicle exit and 5 m sprint layout

For the *rapid patrol vehicle exit* task, a police patrol vehicle (General Motors Holden Commodore SS Sedan) was parked so that the driver’s side of the vehicle opened onto a track. Officers were seated in the driver’s seat of the vehicle without their seatbelt on and with both hands on the steering wheel. A researcher gave a verbal command ‘go’ to start the scenario whilst simultaneously breaking the beam of the electronic timing system light gate (Smart Speed, Fusion Sport, Australia). The officer exited the driver’s side of the vehicle and ran to the rear of the vehicle through two opposing timing gates placed 5 m away, as measured using a digital mini-measuring wheel (Senshin Industry Co., Ltd. Osaka: Japan) (see Fig. 1 below). The task was completed as quickly as possible with officers being given only one opportunity to complete this scenario. As soon as officers completed the activity they were asked to rate, using the VAS, how the ILAV or normal station wear they were wearing impacted on their movement during the task.

The marksmanship task required the officers engage a Z4 target (human silhouette live fire target with four scoring zones) with a Glock model 22 pistol, firing 26 rounds in total, with marksmanship scored over three separate sequences. These three sequences were: Point/proximity shooting (9 rounds); Immediate distance / kneeling (5 rounds); and Transition drills / reloading (12 rounds). Each sequence assessed a single or related multiple skill set which was deemed a mandatory and necessary skill set for the operational policing environment. As soon as officers completed their marksmanship scenarios they were asked to rate on the VAS how the ILAV or normal station wear they were wearing impacted on their shooting ability. For the duration of the marksmanship task, the volunteer officers were under the direction and authority of the State police force Range Safety Officers.

### Subjective evaluation of ILAV

**Fig. 2:**
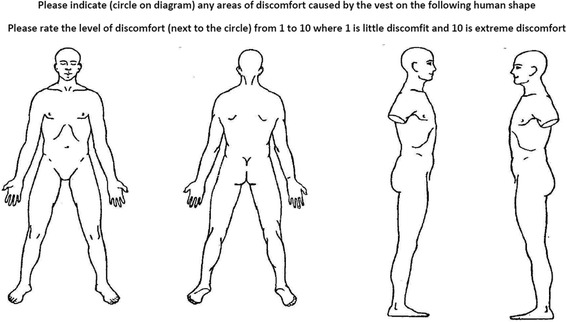
Handout given to officers at the end of each day to enable them to subjectively describe and depict their experience of their daily load carriage configuration

The participating officers were instructed not to remove their assigned ILAV, for the duration of each day (e.g. during lunch or short breaks), and following completion of activities on each day, the officers were requested to indicate on a manikin figure (Fig. 2) any areas of discomfort they felt whilst wearing their assigned ILAV or normal station wear.

### Data analysis

All recorded data except for data relating to the participants’ subjective evaluations of ILAV were entered into a spreadsheet in SPSS version 23 (IBM 2015) and were then cleaned for analysis. Initial descriptive analyses were conducted to provide counts, means, standard deviations and ranges for the included variables, as relevant depending on levels of measurement. These descriptive statistics were derived for each ILAV type and for the normal station wear.

Following these descriptive analyses, a multivariate repeated measures analysis of variance (ANOVA) was conducted to examine the relative impacts of the different ILAV types and normal station wear, with post hoc pairwise comparisons using a Bonferroni adjustment. Finally, subjective evaluations documented by the participants regarding comfort of the ILAV and normal station wear were aggregated onto a single body chart for each type of ILAV assessed, in order to provide an overview for each ILAV type of the evaluations from all participants. Comments on each body chart were numbered according to the identification number assigned to the participant who made the comment, so that multiple comments by the same participant could be identified and so that where comments were more frequent from particular participants, commonalities in those participants’ characteristics (e.g. height, weight or chest circumference) that might have affected their evaluations could be considered.

## Results

### Body armour weights

An overview of the measured weights of each ILAV type is provided in Table 2, where it is apparent that mean weights varied between ILAV types by 0.3 to 0.9 kg and maximum weights (reflecting the largest sizes) varied between ILAV types by 0.7 to 1.5 kg, indicating differences of probable practical or operational significance. All of the differences in mean weights between the three types of ILAV depicted in Table 3 reached statistical significance (*p* < .04 in all instances).

**Table 3:** Means, standard deviations (SD) and ranges of measured weights of each type of ILAV

ILAV type	Mean weight (kg)	SD (kg)	Minimum (kg)	Maximum (kg)
A	4.12	0.65	3.52	5.50
B	3.54*	0.70	2.90	4.82
C	3.24*‡	0.48	2.54	4.04

* Significantly different (*p* < .001) from ILAV A: ‡ Significantly different (*p* < .001) from ILAV B

### Subjective impact of ILAV type on victim drag task effort

Subjectively, ILAV type significantly affected required effort in the victim drag, as assessed by participants using the RPE scale (F [3,30] = 2.964, *p* = .048). Average RPE scores attributed to the victim drag task by officers when wearing each type of ILAV are seen in Table 4 below. These results suggest that officers perceived the victim drag task to be easier to complete when wearing ILAV B than when wearing ILAV A or C or normal station wear. The subjective preference for ILAV B was mirrored in the performance results for the task, with ILAV B being associated with the lowest average time for the task, at 5.47 ± 0.87 s, followed by ILAV C (5.50 ± 1.06 s), station wear (5.56 ± 0.85 s) and then ILAV A (5.74 ± 0.94 s).

**Table 4:** Results for each task. Subjective ratings expressed as mean (95% CI), Task performance results expressed as mean ± SD

	ILAV A	ILAV B	ILAV C	N
Victim Drag				
RPE	4.7(3.6–5.8)	3.6 (2.6–4.7)	4.0 (2.8–5.2)	3.8 (2.4–5.3)
Time to complete	5.74 ± 0.94 s	5.47 ± 0.87	5.50 ± 1.06 s	5.56 ± 0.85 s
Vehicle Exit				
Subjective Rating	−3.58 (−6.0 to −1.1)	+ 0.26 (−2.1 to + 2.5)	−0.55 (− 1.8 to + 0.8)	−0.85 (−4.7 to + 3.0)
Time to complete	3.49 ± 0.28	3.41 ± 0.23	3.40 ± 0.38	3.41 ± 0.43
Range Shoot				
Subjective Rating	−2.1 (−5.5 to + 1.3)	+ 2.7 (+ 0.4 to + 5.0)	− 1.7 (−4.4 to + 0.9)	+ 1.4 (− 2.2 to + 5.0)
Score	80.73 ± 12.25	85.64 ± 7.04	81.45 ± 9.06	83.82 ± 11.20

### The effect of ILAV on vehicle exit and 5 m Sprint

ILAV B was, on average, rated by participants as *aiding* in the performance of the vehicle exit and 5 m sprint task, whereas other load configurations were perceived to have a negative impact on performance of this task. The mean individual VAS scores for participant-perceived impact of the ILAV conditions on task performance were positive for ILAV B and negative for all other conditions, including normal station wear (Table 4).

These results indicate that the officers perceived that ILAV B improved their performance on the vehicle exit and 5 m sprint scenario, whereas they perceived that ILAB A and C and even station wear negatively affected their performance in this particular scenario. The negative mean VAS for ILAV A aligned with the slowest time of the scenario, with an average time of 3.49 ± 0.28 s, but there were minimal time differences between ILAV B, ILAV C or station wear (3.41 ± 0.23 s, 3.40 ± 0.38 s and 3.41 ± 0.43 s respectively).

### Officer-perceived impact of each type of ILAV on range shoot task performance

The repeated measures ANOVA indicated that ILAV condition significantly affected officer self-assessments using the VAS of ILAV impacts on task performance (marksmanship) during the range shoot (F [3,30] = 3.57, *p* = .026). The average impact officers perceived each ILAV condition to have on their performance in the range shoot, as measured by the VAS, was positive in both ILAV B and normal station wear and negative in ILAV A and ILAV C (Table 4).

These results indicate that officers perceived that wearing ILAV B *improved* their performance on the range shoot, even more than wearing the less restrictive and lighter normal station wear, whereas they perceived ILAV A and ILAV C to have significant negative impacts on their range shoot performance. The positive subjective perceptions associated with both ILAV B and normal station wear were reflected in the actual scores of the marksmanship task, with ILAV B associated with an average score of 85.64 ± 7.04 points and station wear 83.82 ± 11.20 points. The negative subjective ratings for both ILAV A and ILAV C were also reflected in the marksmanship results, with ILAV A associated with an average score of 80.73 ± 12.25 points and ILAV C 81.45 ± 9.06 points.

### Subjective evaluations of ILAV types by officers

**Fig. 3:**
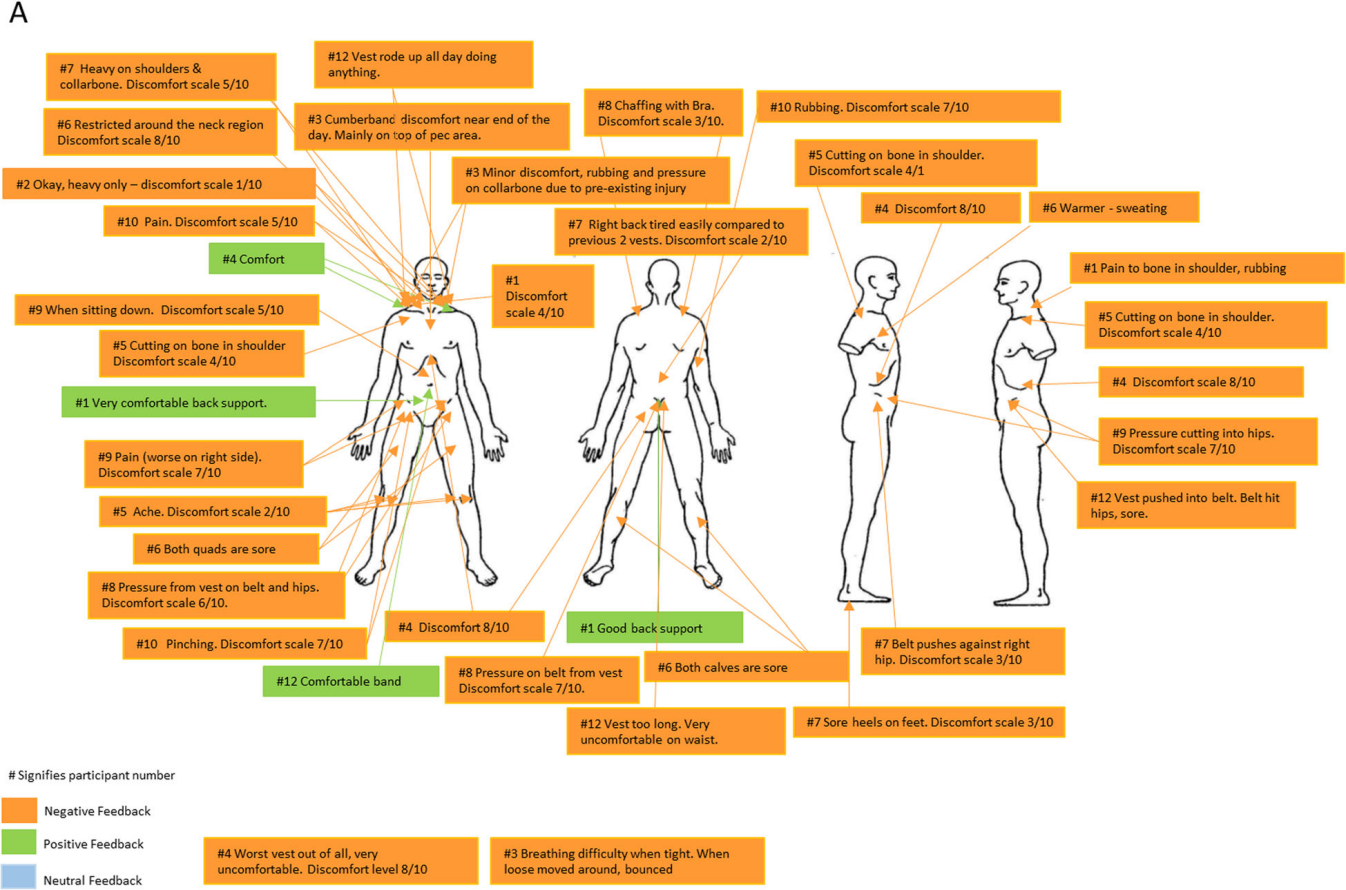
Subjective Evaluation of ILAV A

**Fig. 4:**
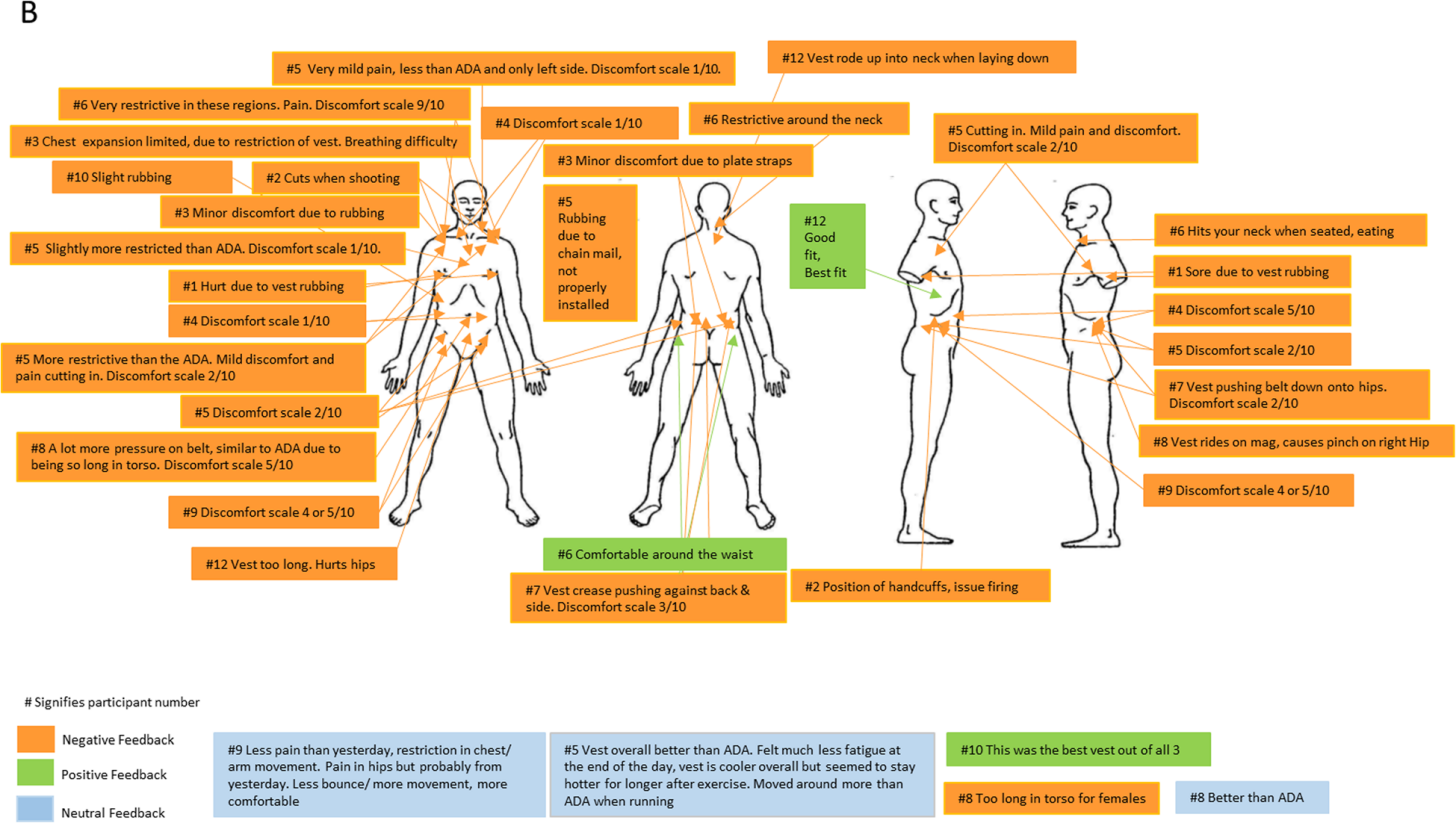
Subjective Evaluation of ILAV B

**Fig. 5:**
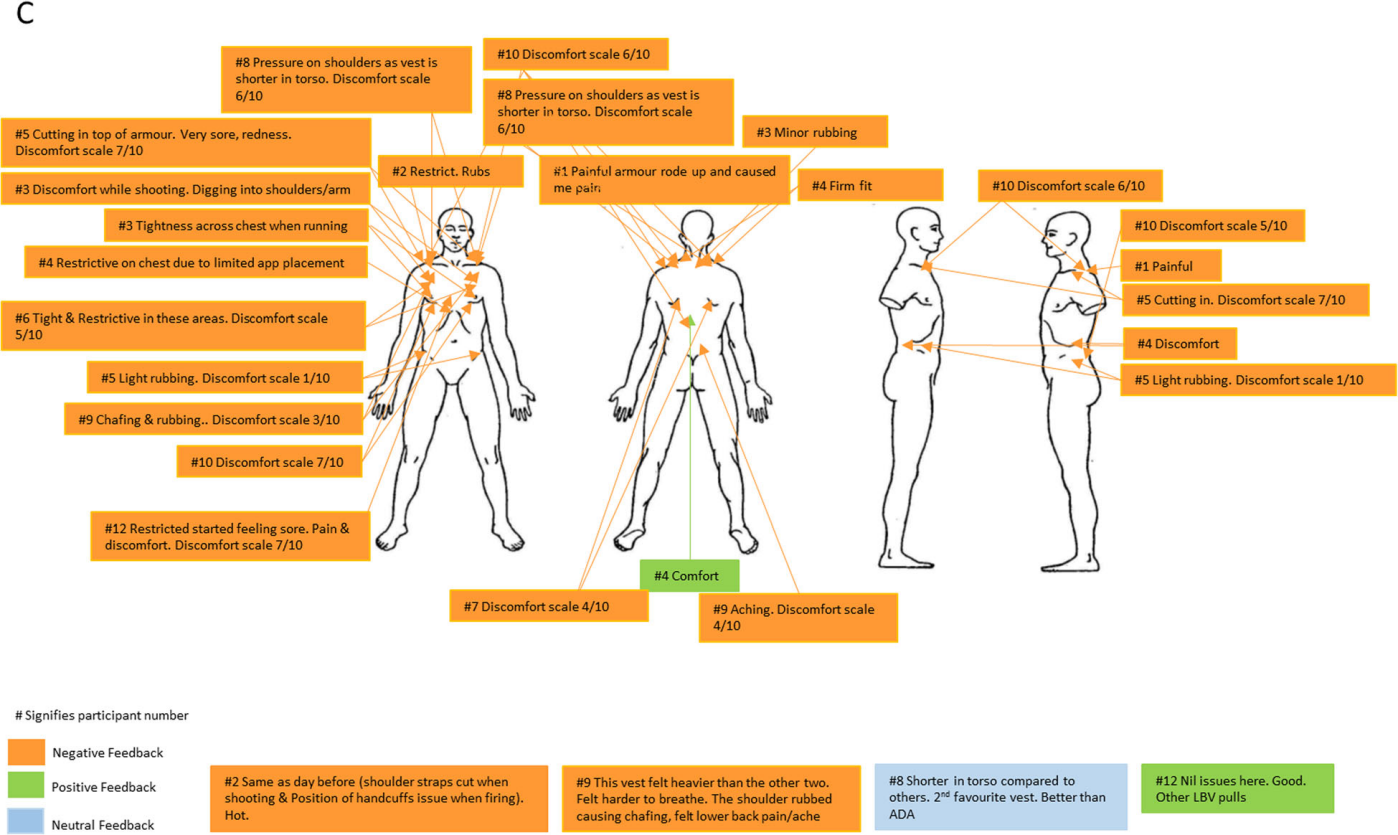
Subjective Evaluation of ILAV C

**Fig. 6:**
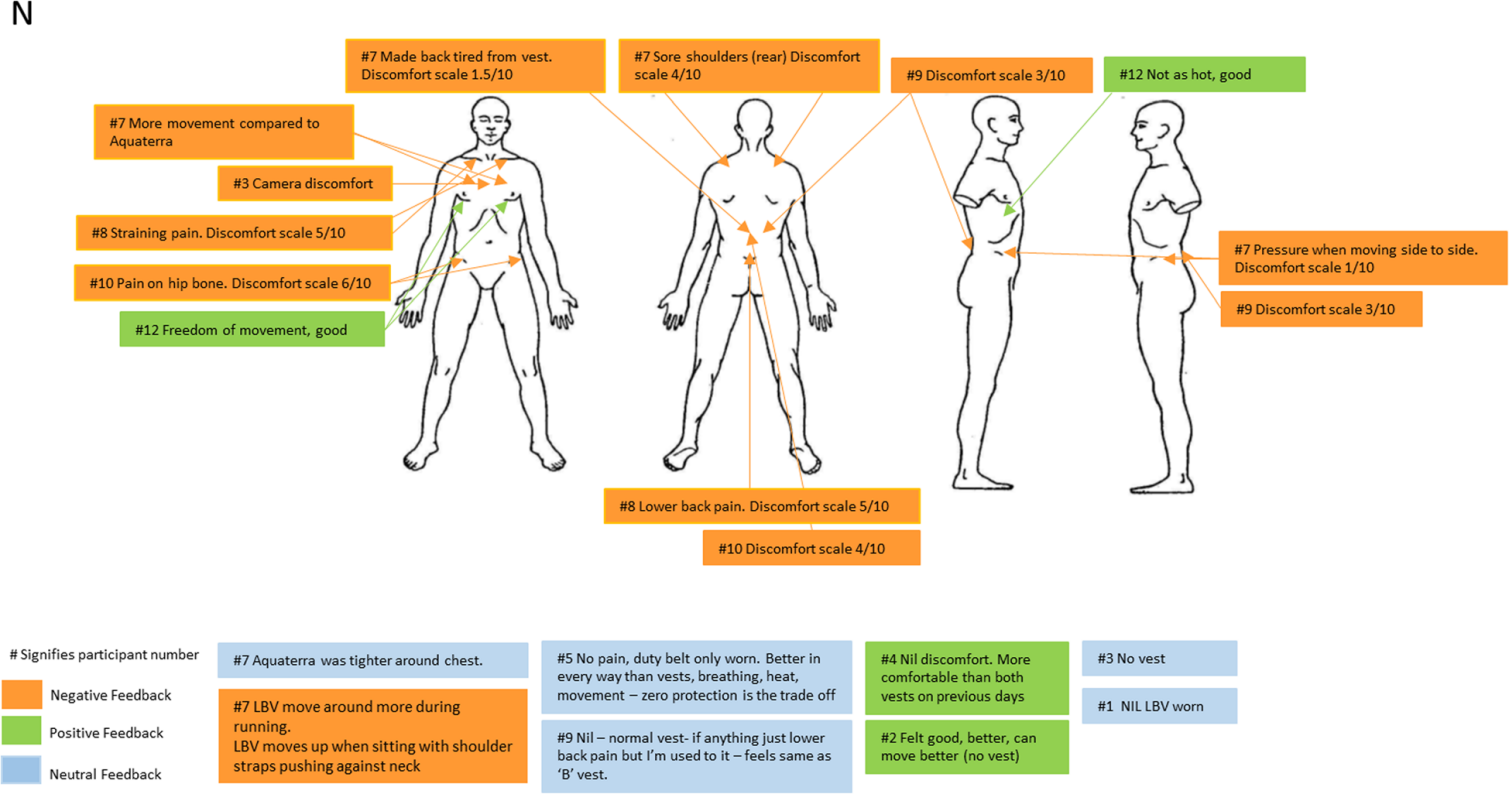
Subjective Evaluation of normal station wear

Figures 3, 4, 5 and 6 provide details of the feedback officers provided regarding their perceptions of the comfort of each type of ILAV. Although all ILAV types and even normal station wear received negative feedback for the discomfort they caused for some participants, it appears that ILAV B received lower ratings of discomfort overall, ranging from 1 to 5 out of 10, with only one exception which appears to have been due to an excessively tight fitting ILAV. Three separate officers specifically noted that ILAV B was more comfortable than ILAV A and a further officer noted that ILAV B was better than both ILAV A and ILAV C. Overall, ILAV B received more favourable comments than either of the other two types of ILAV, though not as favourable as comments regarding normal station wear.

The main complaints with ILAV B, which came from several officers, were that it was long in the torso, causing discomfort and pressing down on handcuffs, magazines, belts and hips. However, the fact that it was longer and sat on the hips seemed to relieve pressure on the shoulders and in contrast, officers complained that body armour ILAV C was too short and therefore put excessive pressure, causing substantial discomfort, on the shoulders and neck, despite the fact it was a lighter type of body armour.

While it seems to have been the most comfortable type of body armour for most officers, consideration should be given to the possible operational impacts of ILAV B, given it was long and, according to participants, placed pressure on the belt, hips, and key police equipment, potentially interfering with officer mobility and ready deployment of key police equipment.

## Discussion

The aim of this investigation was to determine the impacts of three Individual Light Armour Vests (ILAV) and normal station wear on subjective measures of performance and comfort whilst police officers performed occupational tasks associated with their normal policing duties. Despite numerous previous investigations reporting the effects of wearing armour on performance and mobility, there has been minimal focus on the opinion of the individual who wears the armour and no known studies comparing multiple types of ILAV.

There were significant differences in the weights of the three types of body armour used in this study, with ILAV A being the heaviest and ILAV C being the lightest. Also of note, body armour weight increased as the body weight, height and chest circumference of the officer increased, since the size of the body armour required to fit them increased and so also its weight.

The results from this study suggest that overall, ILAV B appeared to be the most preferred by officers when compared to other ILAV configurations, across all three tasks. For both the 10 m victim drag and marksmanship, ILAV B received significantly better ratings that the other ILAV configurations in relation to its impact on task performance. For the 10 m victim drag task the least amount of effort required to complete the task across all ILAV conditions was reported by participants to be associated with ILAV B. Likewise, ILAV B received positive (ie beneficial impact) ratings regarding impacts on marksmanship performance. Interestingly, the quickest times of the Victim drag and the best score on the marksmanship task were whilst wearing ILAV B, so officer perceptions matched objective measures of performance on those tasks.

In contrast to previous research [5–7, 16, 17] highlighting the negative effects of armour, and rather unexpectedly, ILAV B alone had positive impacts on officer perceptions of victim drag task effort, on officer perceptions of the impact of the worn configuration on their marksmanship performance and on victim drag and patrol vehicle exit task performance, though not all of these findings reached statistical significance. It is possible that the improved marksmanship observed and perceived by officers with ILAV B when compared to normal station wear may have been due to a stabilization effect caused by the ILAV over the shoulders [18]. However, improved performance would then also be expected from other ILAV conditions, and this did not occur. In these instances, the discomfort reported by officers for these other ILAVs may have either indirectly (i.e. distracting the officer) or directly (e.g. differing shoulder loading impacts) impacted on the marksmanship performance.

When data from each configuration’s subjective evaluation sheet was collated, it was apparent that the major areas of discomfort for all ILAV were the chest, shoulders and hips. This is in agreement with previously published research which found that, apart from the foot, the low back and hips were the areas of least comfort for carriers of load [17, 19, 20]. However, ILAV B received more favourable comments, and was perceived to have more positive impacts than normal station wear and either of the other two types of body armour, with the main complaints from several participants regarding ILAV B being that it was long in the torso, causing discomfort and pressing down on the hips and equipment placed in this area (e.g. handcuffs, magazine, belt, etc). Previous research has demonstrated that females experience significantly greater discomfort than males around the hips during load carriage tasks [17]. Considering this, modifying the ILAV B to decrease plate or overall design torso length may remove load from the hips and reduce complaints in that area, but it is possible that making this change might shift load to the chest and shoulders; noting that these areas were the key sites of discomfort reported for the other ILAVs.

The outcomes of this study demonstrate that the subjective feedback of officers regarding both comfort and performance are important to consider for the issuing of ILAV and that their perceptions regarding performance are accurate. For example, there was a notable preference for one of the three types of ILAV and whilst wearing this preferred ILAV, the subjective perceptions of performance were in most cases reflected in the objective measures of performance in the tasks.

The future development and large-scale implementation of ILAVs should ensure that the user’s feedback and opinion is sought to ensure that first and foremost they are going to be worn, and that perceived comfort and performance impacts are considered. Seeking the opinion and acceptance of the officers who will be required to wear the equipment is vital to ensure a balance between user acceptance and equipment requirements are obtained, and given the costs associated with the procurement of new equipment for law enforcement, studies on subjective acceptance should be performed before any procurement decisions are made.

### Limitations

There are some limitations to this study which need to be acknowledged. The results from this study are only applicable to the 3 variants which were utilised in this study and therefore may not be representative of all options available to law enforcement officers. The opinions expressed by the sample of officers in this study may also not be representative of the entire police force and a larger scale study may therefore be warranted.

## Conclusions

The results from this study suggest that participants perceived that wearing ILAV B assisted them in performing several key occupational tasks (marksmanship, victim drag and vehicle exit). The ILAV B also had fewer negative comments with respect to comfort and fit throughout the testing period and these findings may at least in part have been due to the longer torso design of ILAV B, shifting load of the ILAV from shoulders and chest to the hips and pelvis. Conversely, the other ILAVs were found to be more uncomfortable on the neck and shoulders, and detrimental to occupational task performance, when this was assessed both subjectively and also objectively. Nevertheless, ILAV B did cause more pressure on hips and pelvis and obstructed deployment of belt-mounted tactical equipment to some degree, according to participants, and this may be of operational concern. Overall, the results from this study suggest that feedback from wearers is important when considering the implementation of ILAV and involving personnel in this way may form an important part of organisational acceptance of new ILAV.

## Abbreviations

ILAV: Individual Light armour vest
N: Normal station wear
PRE: Perceived rate of exertion
RPE: Rating of perceived exertion
VAS: Visual analogue scale
